# Imaging of Pulmonary Miliary Tuberculosis With Multiple Intracranial Tuberculomas

**DOI:** 10.7759/cureus.70373

**Published:** 2024-09-28

**Authors:** Gaurav V Mishra, Anurag Luharia, Shreya Khandelwal, Manasa Suryadevara, Souvik Sarkar, Anshul Sood

**Affiliations:** 1 Radiodiagnosis, Datta Meghe Institute of Higher Education and Research, Wardha, IND; 2 Radiological Safety, Datta Meghe Institute of Higher Education and Research, Wardha, IND; 3 Respiratory Medicine, Datta Meghe Institute of Higher Education and Research, Wardha, IND

**Keywords:** brain lesion, contrast-enhanced mri, gene xpert, miliary, mycobacterium tuberculosis, ptb, radiology, tuberculoma, tuberculosis

## Abstract

Tuberculosis, caused by *Mycobacterium tuberculosis*, is a widely spread disease complex affecting multiple organs. It is a type of communicable disease disproportionately affecting low and middle-income countries. The imaging modality of choice for pulmonary tuberculosis is computed tomography, and for brain lesions, it is a contrast-enhanced magnetic resonance imaging study. This report presents the case of a 73-year-old male patient who was diagnosed with tuberculosis on radiography and was started anti-tubercular treatment for the same and later developed multiple tuberculomas. This report showcases the imaging findings and emphasizes the need for timely and undisrupted treatment for tuberculosis management to prevent further complications like brain tuberculomas as developed in our case.

## Introduction

Tuberculosis (TB), caused by *Mycobacterium tuberculosis*, is the most common communicable disease of the lungs, which can also be disseminated to numerous extrapulmonary sites, including bones, meninges, bowel, adrenal glands, etc., via hematogenous spread [1]. Pulmonary miliary TB is due to the hematogenous spread of the primary focus in the lungs and appears as small diffusely scattered nodules (2-3 mm), giving the appearance of millet seeds [2]. Sometimes, the size of the nodules may be large, as seen in our case. CNS TB may be intra-axial, which may manifest as tuberculoma, tubercular abscess, miliary TB, or extra-axial, which may manifest as tubercular meningitis or pachymeningitis (rare) [3].

## Case presentation

**Figure 1:**
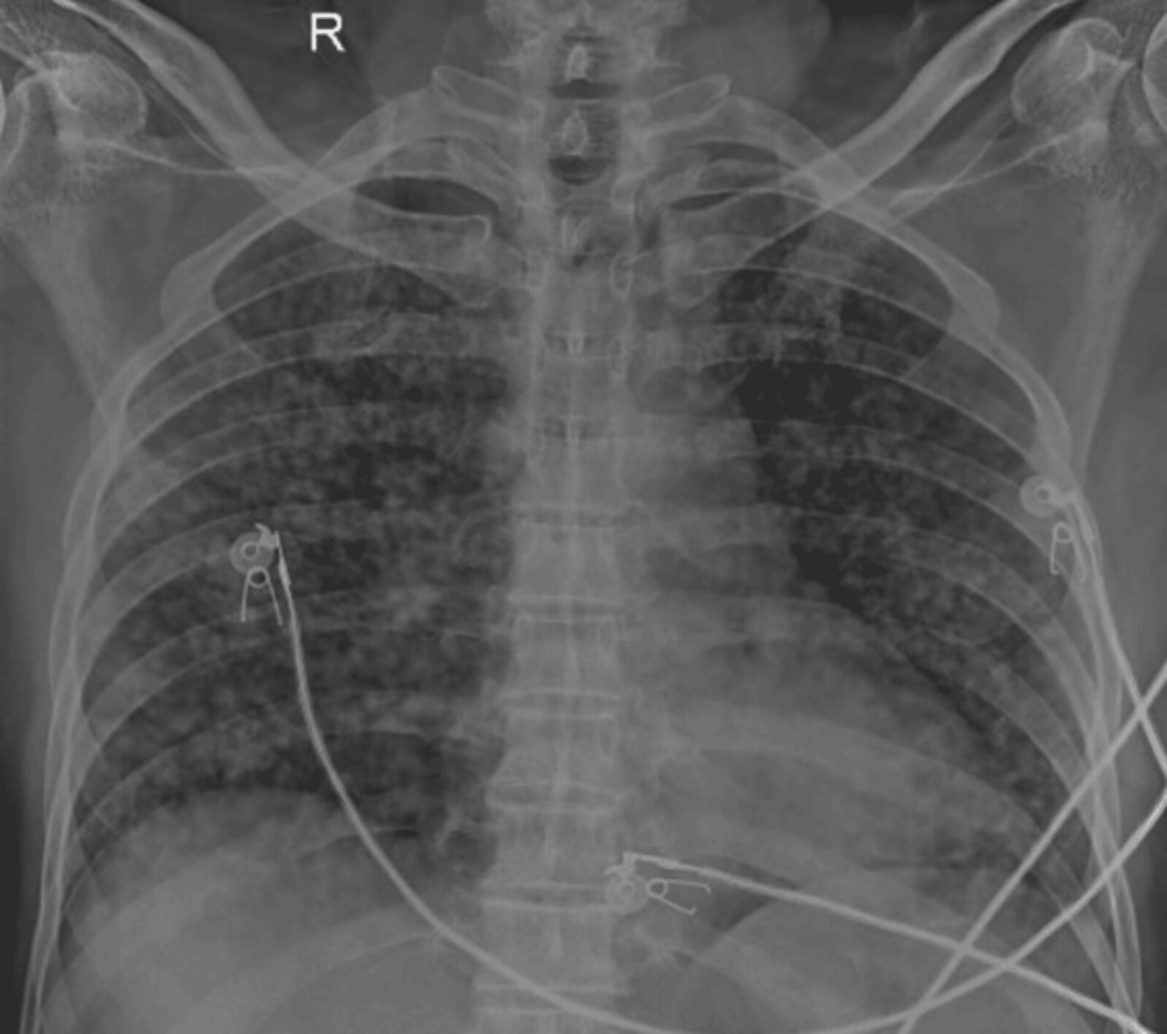
Chest radiograph showing diffusely scattered multiple tiny nodules in bilateral lung fields suggesting miliary tuberculosis.

A 73-year-old male patient presented to the respiratory outpatient department (OPD) with complaints of breathlessness and an evening rise in temperature for one month. The patient gave a history of his wife having pulmonary TB 10 years ago, which was completely treated by an anti-tubercular drug regimen for six months. Chest X-ray revealed the presence of diffusely scattered multiple tiny nodules in bilateral lung fields (Figure 1) with a positive sputum test on GeneXpert® (Cepheid, Sunnyvale, California, United States) cartridge-based nucleic acid amplification test and Ziehl-Neelsen (ZN) staining for acid-fast bacilli (AFB) (+3).

**Figure 2:**
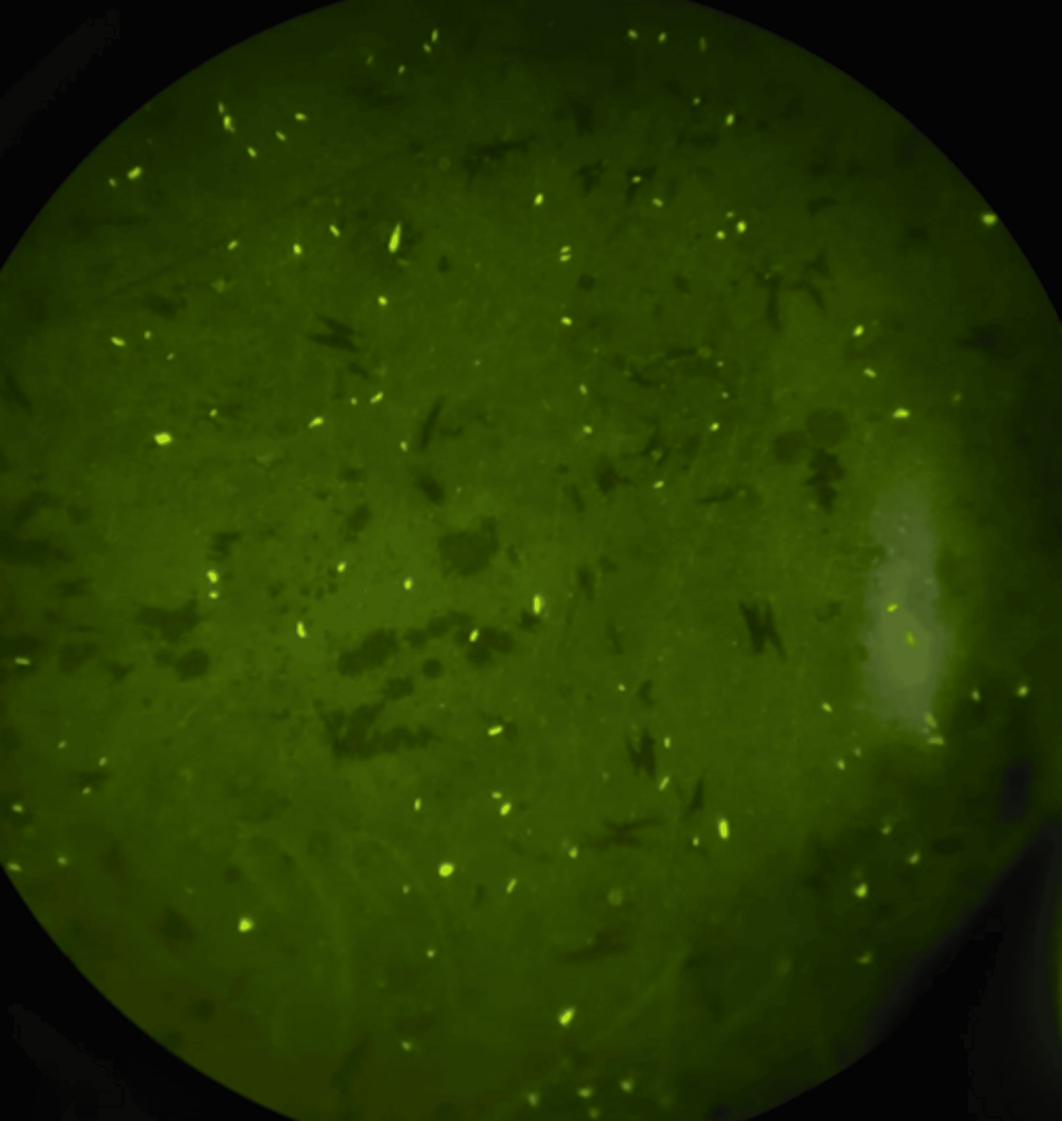
Fluorescent microscopy showing multiple acid-fast bacilli.

The sputum was also positive on fluorescent microscopy for AFB (Figure 2).

The patient was then started on an anti-tubercular treatment comprising isoniazid, rifampicin, pyrazinamide, and ethambutol.

**Figure 3:**
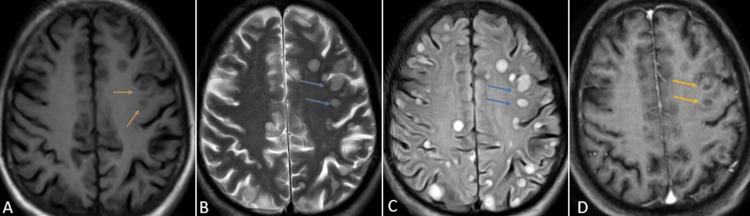
CE-MRI of the brain axial sections at the level of the centrum semiovale T1WI (A), T2WI (B), FLAIR (C), and T1 post-contrast (D) sequences showing variably sized, diffusely scattered round to oval lesions appearing hypointense on T1WI (orange arrows), hyperintense on T2WI/FLAIR (blue arrows) with post-contrast ring enhancement (yellow arrows) suggesting tuberculomas. CE-MRI: contrast-enhanced magnetic resonance imaging; FLAIR: fluid attenuation inversion recovery; T1WI: T1-weighted imaging; T2WI: T2-weighted imaging

**Figure 4:**
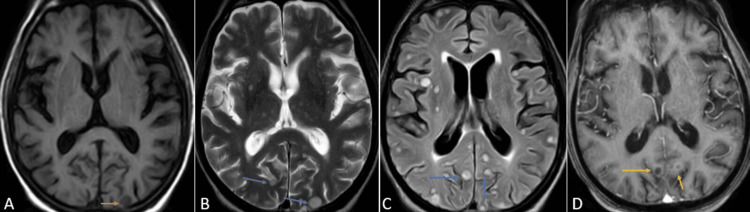
CE-MRI of the brain axial sections at the level of the ganglion-capsular region T1WI (A), T2WI (B), FLAIR (C), and T1 post-contrast (D) sequences showing variable sized, diffusely scattered round to oval lesions appearing hypointense on T1WI (orange arrows), hyperintense on T2WI/FLAIR (blue arrows) with post-contrast ring enhancement (yellow arrows) suggesting tuberculomas. CE-MRI: contrast-enhanced magnetic resonance imaging; FLAIR: fluid attenuation inversion recovery; T1WI: T1-weighted imaging; T2WI: T2-weighted imaging

**Figure 5:**
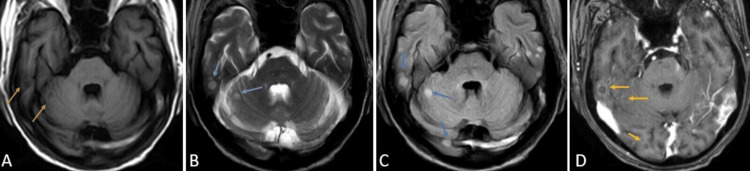
CE-MRI of the brain axial sections at the level of the cerebellum T1WI (A), T2WI (B), FLAIR (C), and T1 post-contrast (D) sequences showing variable sized, diffusely scattered round to oval lesions appearing hypointense on T1WI (orange arrows), hyperintense on T2WI/FLAIR (blue arrows) with post-contrast ring enhancement (yellow arrows) suggesting tuberculomas. CE-MRI: contrast-enhanced magnetic resonance imaging; FLAIR: fluid attenuation inversion recovery; T1WI: T1-weighted imaging; T2WI: T2-weighted imaging

One month later, the patient was brought to the emergency department of the hospital by relatives with complaints of slurring of speech and weakness in all four limbs for the past three days. Upon speaking with the relatives, it was concluded that the patient did not take the medications as prescribed. Contrast-enhanced magnetic resonance imaging (CE-MRI) of the brain was performed, which revealed variable-sized, diffusely scattered round to oval lesions appearing hypointense on T1 weighted imaging (T1WI), hyperintense on T2 weighted imaging (T2WI) and fluid attenuation inversion recovery sequence (FLAIR) with post-contrast ring enhancement suggesting tuberculomas in the bilateral cerebral hemisphere and cerebellum (Figures 3-5).

A workup of the patient was conducted, which included blood tests and high-resolution computed tomography (HRCT) of the chest. Lab parameters of the patient are shown in Table 1.

**Table 1:** Lab investigations of the patient. MCHC: mean corpuscular hemoglobin concentration; MCV: mean corpuscular volume; MCH: mean corpuscular hemoglobin; HCT: hematocrit; RDW: red cell distribution width

Parameters	Patient’s value	Reference values
Hemoglobin	12.4	12.1-15.1 g/dl
MCHC	33.9	32-36 g/dl
MCV	81.5	80-100 fl
MCH	27.7	27-33 pg
Total RBC count	4.47	4.3–6.1 cells/mcl
Total WBC count	19500	4000-9000 g/dl
Total platelet count	320000	150000-400000 g/dl
HCT	36.5	36-44%
RDW	15.7	12.2-16.1%
Urea	88	5-20 mg/dl
Creatinine	2.1	0.6-1.1 mg/dl

**Figure 6:**
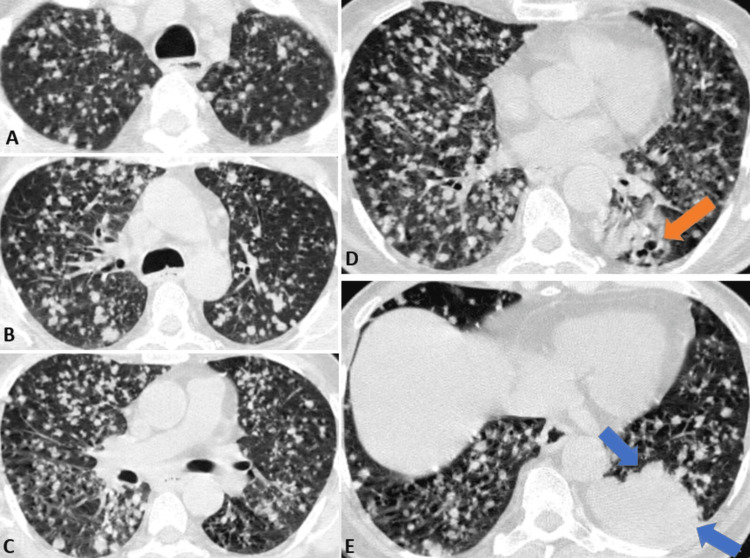
HRCT image of the chest axial sections showing diffusely scattered nodules in the bilateral lung parenchyma at the mid-tracheal level (A), carina (B), sub-carinal level (C) with a patchy area of consolidation in the left lower lobe (E) (blue arrow) with air-filled bronchi surrounded by fluid-filled alveoli (D) (orange arrow) suggesting miliary tuberculosis. HRCT: high-resolution computed tomography

HRCT of the lungs revealed multiple diffusely scattered nodules in the bilateral lung fields, the largest measuring approximately 7 mm in diameter (Figure 6).

Lumbar puncture was also done, which revealed a positive GeneXpert and ZN staining for AFB. The proteins were high, measuring 0.3 g/L, low sugar level, measuring 2 mmol/L, and a predominance of lymphocytes. The white blood cell count was 420 cells/microliter.

The patient was given an injection of dexamethasone 8 mg thrice daily along with anti-tubercular drugs. Tablet levetiracetam 500 mg was given twice daily prophylactically. There was a decrease in the sodium levels, measuring 115 Meq/L, and was treated using sodium supplements. Once stable, the steroid was gradually tapered, and the patient was discharged with advice to continue the anti-tubercular drug treatment as prescribed and to follow up after 15 days.

## Discussion

Pulmonary miliary TB can be seen in both primary and post-primary TB and is caused by hematogenous dissemination of the primary complex throughout the lungs. The mycobacterium focus can reach the CNS via hematogenous spread by crossing the blood-brain barrier and can manifest as tuberculoma, tubercular abscess, and meningitis [4,5].

The first imaging modality of choice is chest radiography, and computed tomography (CT) is the imaging modality of choice for pulmonary TB. The appearance of miliary tuberculosis on CT is diffusely scattered, evenly distributed small nodules (2-3 mm) in bilateral lung fields, giving the appearance similar to that of millet seeds; hence, the name miliary tuberculosis. Additionally, findings of parenchymal consolidation and lymphadenopathy may be present. Macro nodules may be seen, which are due to the confluence of several air space nodules or granulomas [6].

CE-MRI is the imaging modality of choice for diagnosing brain lesions. Miliary TB may appear diffusely scattered, measuring less than 2-3 mm in the grey-white matter junction with homogenous post-contrast enhancement [4]. Tuberculomas may show homogenous or ring enhancement with no diffusion restriction, while the tubercular abscess will show ring enhancement with areas of diffusion restriction [7]. Rarely does the CNS spread have extra-axial manifestations in the form of pachymeningitis, which appears as a homogenous enhancement of the thickened meninges [8,9].

The differential diagnosis for pulmonary miliary TB includes miliary metastatic deposits, which are most commonly seen in cases of lung, breast, or thyroid carcinoma [1,10]; Langerhans histiocytosis appearing as a combination of cysts and nodules and silicosis or coal worker pneumoconiosis, which are strongly associated with exposure to coal dust or silica [11].

## Conclusions

TB is very common in developing countries like India. Pulmonary TB is the most common manifestation of the *Mycobacterium* complex. The disease presents with typical signs and symptoms. Early recognition of the signs and symptoms is a must for the early treatment of the disease. HRCT is the imaging modality of choice for imaging pulmonary TB. CE-MRI is the imaging modality of choice for diagnosing brain lesions. Once detected, the disease can be cured with the usage of anti-tubercular regimens if proper dosages are taken without missing the medicines. In cases of missed dosages, TB tends to spread both in the lungs and extrapulmonary region. Therefore, utmost care must be taken to diagnose the disease and complete a drug regimen without missing a dose.
